# Characterization of early phases of cardiomyopathy syndrome pathogenesis in Atlantic salmon (*Salmo salar* L.) through various diagnostic methods

**DOI:** 10.1111/jfd.13659

**Published:** 2022-06-10

**Authors:** Camilla Fritsvold, Aase B. Mikalsen, Øyvind Haugland, Haitham Tartor, Hilde Sindre

**Affiliations:** ^1^ Norwegian Veterinary Institute Ås Norway; ^2^ Faculty of Veterinary Medicine Norwegian University of Life Sciences Ås Norway; ^3^ PHARMAQ Oslo Norway

**Keywords:** cardiomyopathy syndrome, CMS pathogenesis, CMS diagnostic methods, non‐lethal sampling, piscine myocarditis virus

## Abstract

Since the first description of cardiomyopathy syndrome (CMS) in Atlantic salmon, in 1985, the disease caused by piscine myocarditisvirus (PMCV) has become a common problem in Atlantic salmon farming, not only in Norway, but also in other salmon farming countries like Scotland and Ireland. In the last years, CMS has been ranked as the most important salmon viral disease in Norway regarding both mortality and economic losses. Detailed knowledge of infection and pathogenesis is still lacking, a decade after the causal agent was first described, and there is a need for a wider range of methods/tools for diagnostic and research purposes. In this study, we compared the detection of PMCV‐ and CMS‐related tissue lesions using previously used and well‐known methods like histopathology and real‐time RT‐PCR to immunohistochemistry (IHC), a less used method, and a new method, RNAscope in situ hybridization. Tissue samples of three different cardiac compartments, mid‐kidney and skin/muscle tissue were compared with non‐lethal parallel samplings of blood and mucus. The development of pathological cardiac lesions observed in this experiment was in accordance with previous descriptions of CMS. Our results indicate a viremic phase 10‐ to 20‐day post‐challenge (dpc) preceding the cardiac lesions. In this early phase, virus could also be detected in relatively high amount in mid‐kidney by real‐time RT‐PCR. Plasma and/or mid‐kidney samples may, therefore, be candidates to screen for early‐phase PMCV infection. The RNAscope in situ hybridization method showed higher sensitivity and robustness compared with the immunohistochemistry and may be a valuable support to histopathology in CMS diagnostics, especially in cases of untypical lesions or mixed infections.

## 1 INTRODUCTION

Cardiomyopathy syndrome, CMS, is a viral disease of farmed Atlantic salmon, usually affecting larger fish in their second year in sea water. The causative agent piscine myocarditis virus (PMCV) induces severe cardiac inflammation of the spongious layers of the salmonid atrium and ventricle. The disease has been known in Norwegian fish farming since the mid‐1980s and has since then been described in other countries farming Atlantic salmon: Scotland, Faroe Island and Ireland (Poppe & Sande, 1994; T. Poppe & Seierstad, 2003; Rodger & Turnbull, 2000; Rodger et al., 2014), in a few cases of wild Norwegian Atlantic salmon (Poppe & Seierstad, 2003), and a disease resembling CMS has also been described in Canada (Brocklebank & Raverty, 2002). In some cases, the disease period occurs as long‐term, with moderately increase in mortality, but sudden outbreaks with high mortality, often induced by stressful events (Ferguson et al., 2006). Moribund fish are seldom observed. As CMS can cause considerable mortality in large salmon in good condition close to slaughter, the economic impact is significant, and the disease has for several years been ranked as the most important contagious disease causing mortality of Norwegian Atlantic salmon in ongrowing and broodstock sites (Fritsvold & Jensen, 2021; Garseth et al., 2018).

Several hypotheses about the aetiology of CMS were initially discussed, but in 2009, the first experimental challenge experiments succeeded in proving transmissibility of CMS (Bruno & Noguera, 2009; Fritsvold et al., 2009), and in 2010, PMCV was described as the causative agent of CMS (Haugland et al., 2011; Lovoll et al., 2010). Fish with CMS are normally in good condition and may present with macroscopic changes related to circulatory disturbances, that is, exophthalmus, abdominal petechial haemorrhaging and raised scale skin pockets due to oedema. An autopsy can reveal ascites, dark or discoloured liver, often with fibrinous cast on the surface and an enlarged, distended atrium and/or sinus venosus, and in severe cases, a ruptured atrium/sinus venosus and pericardial cavity filled with blood clots (cardiac tamponade). Microscopically, the inflammation is usually restricted to the atrium and spongiosum of the cardiac ventricle, sometimes with epicarditis and/or perivascular or focal myositis in the ventricular compactum (Bruno et al., 2013; Ferguson et al., 2006). PMCV‐specific RNA is usually found in the highest amounts in cardiac tissues, and in lower levels in kidney, spleen and blood (Fritsvold et al., 2021; Timmerhaus et al., 2011).

At present, a CMS diagnosis is usually based on clinical observations, autopsy findings and histopathological examination of cardiac tissue (Fritsvold & Jensen, 2020, 2021). A standard set of samples received for fish diagnostics at Norwegian Veterinary Institute, including CMS, usually contain a selection of formalin‐fixed tissues (gill, heart, liver, exocrine pancreas, mid‐kidney and skin with attached red and white skeletal muscle), in addition to samples of head or mid‐kidney and heart, usually the apex. In some cases, the diagnosis is supported by a real‐time RT‐PCR for PMCV‐specific RNA or occasionally, by an immunohistochemistry (IHC) for PMCV. A histopathological evaluation of other tissues, especially exocrine pancreas, kidney and skeletal muscle, is usually performed, to distinguish CMS from its most important differential diagnosis pancreas disease (PD), heart and skeletal muscle inflammation (HSMI), and to some degree, infectious salmon anaemia (ISA) (Fritsvold & Jensen, 2020).

Initially, CMS was predominantly found in the North‐Western part of Norway, but has since spread and can now be found in all 13 production areas along the Norwegian coast. A similar pattern can be observed for HSMI, while PD is predominantly found in production areas in South‐western‐ to Mid‐Norway. In 2020, there were more than 150 confirmed cases of each of these three viral diseases, all causing cardiac lesions (Fritsvold & Jensen, 2021).

In Norwegian salmonid aquaculture, populations at a fish farm often present with several coexisting infections and diseases, both at site and individual levels. Especially, early stages of the cardiac diseases can be difficult to differentiate histopathologically, but the late, post‐infection cardiac changes of both PD and HSMI can also be challenging when combined with sparse to moderate CMS lesions. This calls for more than histopathological experience and accuracy to distinguish between different stages of potentially several diseases in one individual, and a wider selection of supporting diagnostic tools is required.

An IHC procedure is available for in situ detection of PMCV antigens in relation to the CMS cardiac lesions (Fritsvold et al., 2021), although experiences with the available procedure have shown the method to be less robust and of low sensitivity to be included in routine diagnostics. A traditional in situ hybridization method for the detection of PMCV‐specific RNA has been described (Haugland et al., 2011), but has not been included in routine diagnostics due to a time‐consuming procedure.

Although initial characterization of PMCV described viral replication in a fish cell culture (Haugland et al., 2011), no cell cultures resulting in efficient replication of PMCV to high titres are described or available. Also, commercially available ELISA methods or antibody tests for CMS diagnostics have not been described.

An increasing focus on fish welfare and stronger demands for implementation of the 3Rs (replacement, reduction and refinement) (Tannenbaum & Bennett, 2015; Toni et al., 2019) both in farms and in research makes research on non‐lethal sampling methods like blood sampling, mucosal swabs and water samples relevant, but none of these methods have at present been tested or are available for CMS diagnostics.

Therefore, the main focus of this study was to characterize the initial phases of CMS pathogenesis of Atlantic salmon in a CMS challenge trial, through comparison of histopathological changes and standard real‐time RT‐PCR results of three different cardiac tissues, in addition to mid‐kidney and skin/muscle tissues, with additional results from non‐lethal parallel samplings of blood and mucus. The second goal was to establish a more robust and simple to use in situ method for PMCV for use in CMS diagnostic work and compare this with the immunohistochemistry method for PMCV established at NVI.

## 2 MATERIALS AND METHODS

### 2.1 Experimental fish

Non‐vaccinated Atlantic salmon pre‐smolts reared from eyed eggs at the Industrial and Aquatic Laboratory (ILAB) were used as experimental fish in this challenge experiment. The original batch of eggs originated from StofnFiskur, Egghous Vogavik, Iceland, and their parents had been screened and found negative for SAV, PRV, PMCV, IPNV and ISAV. The eggs hatched early in May 2017, and the fish were start fed 2 months later. To verify that the experimental fish were without histopathological cardiac changes and free of the most common pathogens of Norwegian aquaculture, 60 hearts were examined by light microscopy and gills (*n* = 120), hearts (*n* = 60) and kidneys (*n* = 60) of the fish group tested by PCR for SAV, PRV, PMCV, IPNV, ISAV and SGPV at 5 g size (using a commercial service from Pharmaq Analytiq AS). The fish group was subject to monthly inspections by a fish health biologist, and no disease‐related problems were registered before initiating the challenge. Fish included in this study originated from non‐vaccinated negative controls in an experimental testing of vaccine candidates against CMS (handled by Pharmaq AS). Marking of fish was performed at the start of the challenge in August 2018 by fin‐clipping or shortening of the maxillae. The non‐vaccinated control group described in this study had their left maxillae shortened. Before challenging the test groups, 6 fish were sampled at day 0 and found negative for CMS and PMCV by histopathology and PCR (both at Pharmaq Analytiq AS and NVI).

### 2.2 Facilities and husbandry

The experiment was performed at the Industrial and Aquatic Laboratory (ILAB, Stiftelsen Industrilaboratoriet), in Bergen in early autumn 2018. The 45 fish were kept at 12°C in 500 L tanks of freshwater with in total of 225 additional experimental fish, and fish and tanks were tended and monitored on a daily basis by facility personnel. The fish were fed according to appetite throughout the study and were taken off feed for a minimum of 24 h prior to i.p. injection of mock vaccine (see Challenge chapter) and before challenging. Moribund fish were to be killed and logged as mortalities, and remarks were to be made if challenged fish displayed typical or atypical signs of disease. Abnormal or unexpected behaviour, loss of appetite, unexpected mortality or signs of disease were to be reported immediately. The in vivo study was conducted in compliance with approval 15,581 issued by the Norwegian Food Safety Authority.

### 2.3 Preparation of challenge material and challenge

The fish were intraperitoneally (i.p.) injected with a mock vaccine (PBS) 7 weeks before challenging. Challenge material was prepared from spleen tissue collected from moribund Atlantic salmon (*Salmo salar* L.) from a field outbreak of CMS (diagnosis confirmed by histopathological examination). In total, 10 mg of tissue was homogenized in Leibowitz L‐15 cell culture media supplemented with gentamycin to a final concentration of 50 μg/ml, using a BMT‐50‐S Tube (IKA) with stainless steel balls in a total volume of 50 ml. The homogenate was made by running the tube on the ULTRA‐TURRAX® Tube Drive for 6 × 1 min at 6000 rpm (1 min on ice between each cycle). Homogenate was thereafter centrifugated at 4000 *g* for 10 min at +4°C, and cellular debris removed before filtered (0.22 m‐filter).

At challenge day, 0‐day post‐challenge (dpc), the fish were i.p. injected 0.1 ml tissue homogenate. The homogenate was found negative by PCR for SAV, PRV and IPNV (Pharmaq) and had a Ct value of 16.5 for PMCV‐specific RNA.

### 2.4 Sampling and preservation

#### 2.4.1 Autopsy

An overview of sampled material at the day of challenge (0 dpc) and at samplings 10, 20 and 52 dpc is found in Table 1. At sampling, fish were sedated, immobilized and anaesthetized with Tricaine Pharmaq® (tricaine mesilate, ‘MS222’) baths. Sedated fish were inspected for any signs of abnormality before mucus swabs were taken, the fish measured, weighed and blood drawn from the caudal vein (*Vena caudalis*), before they were killed by decapitation and other tissues were sampled at autopsy.

**TABLE 1:** Overview of use of sampled fish and material per sampling day post‐challenge (dpc) for histopathology, real‐time RT‐PCR, Immunohistochemistry (IHC) and in situ hybridization (ISH)

Method	Material	Organs/material/location sampled	Individual fish numbers at each sampling			
			Day 0	10 dpc	20 dpc	52 dpc
Histo‐patho‐logy	Tissue	Heart	1–6	1–15	1–15	1–5, 7–11, 13–15^a^
		Mid‐kidney	1–3, 5–6^b^	1–15	1–15	1–11, 13–15^a^
		Skin/muscle	1–6	1–15	1–15	1–11, 13–15^a^
PCR	Tissue	Cardiac				
		Atrium	1	1–15	1–15	1–15
		Spongiosum	1–4	1–15	1–15	1–4^c^
		Compactum	1–4	1–15	1–15	1–15
		Mid‐kidney	1–4	1–15	1–15	1–15
		Skin/muscle	1–4	1–15	1–15	1–15
	Blood	Whole blood	1–4	1–15	1–15	1–15
		Plasma	1–4	1–15	1–15	1–15
		Blood cell pellet	1–4	1–15	1–15	1–15
	Mucus	Mucus from pectoral fin	1–4	1–15	1–15	1–15
		Mucus from lateral line	1–4	1–15	1–15	1–15
		Mucus from anus	1–4	1–15	1–15	1–15
IHC	Tissue	Heart	1, 4	3, 7	2, 5, 12	1, 4
ISH	Tissue	Heart	1, 4	3, 7	2, 5, 12	1, 4
		Mid‐kidney	1	3	2	1

^a^ Fish 6 lacks heart for histology, and fish 12 lacks all three organs for histology.

^b^ Fish 4 lacks mid‐kidney.

^c^ Spongiosum was sampled of the 4 first fish only.

#### 2.4.2 Blood

Blood samples were collected in standard heparinized tubes (‘Vacuette tube’ LH Lithium Heparin, Greiner Bio‐One), placed on a gentle mixer during sampling and stored on ice, before 250 μl of heparinized blood was mixed with 750 μl NucliSENS® (bioMéreux SA) lysis buffer. The remaining blood was pelleted for 10 min at 2500 (0 and 52 dpc) or 3500 (10 and 20 dpc) rounds per minute in a centrifuge. Both 250 μl of the plasma layer and 250 μl of the sedimented blood cells left at the bottom of the tubes were added to separate tubes with 750 μl NucliSENS® each, and left at room temperature for at least 10 minutes before stored at −80°C until analysed.

#### 2.4.3 Mucus

Mucus was collected by rolling three separate sterile cotton swabs around the left pectoral fin, along the lateral line of the left side of the fish and over the anus, respectively. The cotton tip of the swab was then cut off and left in suitable tubes with RLT® lysis buffer (Qiagen) and left at room temperature for at least 10 min before stored at −80°C until analysed.

#### 2.4.4 Tissues

For histological examination and in situ methods, heart, mid‐kidney and skin with underlying red and white muscle of all fish were sampled and fixed in 10% neutral buffered formalin. Subsequently, the samples were prepared by standard procedures (Bancroft & Stevens, 1990), including embedding in paraffin wax and sectioned by cutting approximately 3‐μm‐thick sections placed on slides.

For real‐time RT‐PCR analyses, the three cardiac compartments atrium, ventricular compactum and ventricular spongiosum were sampled separately, in addition to mid‐kidney and skin with underlying red and white muscle, using RNA*later*® (Ambion) for preservation. At the 52 dpc sampling, cardiac spongiosum was sampled of four fish only; hence, cardiac samples of the remaining 11 fish (fish 5–15) consist of atrium and ventricular compactum only at this time point.

### 2.5 Histopathology

The formalin‐fixed and paraffine‐embedded tissue sections were dewaxed and stained with haematoxylin and eosin (HE) after standard procedures (Bancroft & Stevens, 1990).All slides of all organs were examined by light microscopy, unblinded. All hearts were scored from 0 to 4 according to (Bancroft & Stevens, 1990; Fritsvold et al., 2009) (see Table 2), where 0 refers to no pathological lesions and 4 represents severe, typical CMS lesions. Cardiac atrium, ventricular spongiosum and ventricular compactum were evaluated and scored individually for severity and distribution of inflammation, and scale steps of 0.5 were used to increase the resolution of the scoring table.

**TABLE 2:** Histopathological grading of cardiac CMS lesions, according to Fritsvold et al. (2009)

Score	Description
0	No pathological findings, or slightly increased number of leukocytes
1	One or a few focal lesions, increased number of leukocytes
2	Several distinct lesions and small to moderate increase in number of leukocytes
3	Multifocal to confluent lesions and moderate to severe increase in number of leukocytes
4	Severe confluent lesions comprising >75% of the tissue and massive leukocyte infiltration

### 2.6 Extraction of RNA and real‐time RT‐PCR

Purified total RNA was extracted from the sampled material using a MagNA Pure 96 (Roche) high‐throughput robotic workstation following the recommendations from the manufacturer. For the heart and kidney samples, a piece of tissue (≤20 mg) was homogenized in 600 μl MagNA Pure lysis buffer. The blood samples and mucus swabs, collected on lysis buffer, were used directly. For extraction, 500 μl of each sample in lysis buffer was used as input with resulting 50 μl eluate. The RNA concentration was then measured on a NanoDrop 8000 (ThermoFisher Scientific) for the heart and kidney samples, and 500 ng RNA was used as input for real‐time RT‐PCR. A fixed amount of 7.5 μl of extracted RNA eluate was used as input for real‐time RT‐PCR of the mucus swabs and blood samples, to compare the amount of viral RNA per volume of these samples. Real‐time RT‐PCR was performed with primers and probe targeting a conserved area of the RNA‐dependent RNA polymerase gene (ORF2) of PMCV, as described in (Lovoll et al., 2010). All samples of each sampled material were analysed in a single run.

### 2.7 Immunohistochemistry

A PMCV‐specific immunohistochemistry (IHC) protocol employing a polyclonal antibody towards ORF3 was performed on a selection of 9 heart sections from the samplings 10, 20 and 52 dpc, as described previously with some modification (Fritsvold et al., 2021) with some modification. In summary, dewaxed and dehydrated 3 μm slides of cardiac tissue were incubated for 20 min at room temperature with 5% BSA in a Tris buffer, followed by a 120‐min incubation with a 1:2000 primary rabbit polyclonal antibody dilution, based on recombinant proteins from ORF3 (ΔORF3, ZN01101, Rabbit 062, PMCV), kindly donated by M. Rode, Pharmaq AS. Then, the slides were incubated for 30 minutes with a 1:500 secondary antibody dilution (biotinylated goat anti‐rabbit Ig. DAKO E 432; DAKO), before another 30‐minutes incubation, with Streptavidin alkaline phosphate in a 1:500 dilution (Streptavidin‐AP, Vector SA‐5100), final visualization with Fast Red. Negative control samples of cardiac tissue from healthy Atlantic salmon, known negative to PMCV, PRV and SAV by real‐time RT‐PCR were included, and as a positive control, a heart from a clinical field outbreak of CMS with severe lesions and high PMCV‐specific RNA load was included (Fritsvold et al., 2021).

### 2.8 In situ hybridization

An RNAscope in situ hybridization assay targeting PMCV was established and used for comparison to confirm the results of PMCV load indicated by the real‐time RT‐PCR. The results were also used to study viral tropism in infected tissues and in relation to cardiac lesions if present, and for comparison with IHC detecting PMCV antigen in serial tissue sections of hearts. Slides were made of 9 selected formalin‐fixed cardiac tissue samples, representing all four sampling time points (for details, see Table 1). The selection of these samples was based on their cardiac PCR results, to ensure the inclusion of individuals with the highest amounts of PMCV‐specific RNA; hence, the samples are not representative for all samples at each sampling point. In addition, slides of mid‐kidney from one of the fish at each sampling point represented by a cardiac sample were included.

For this purpose, the RNAscope® 2.5 HD Singleplex Red Chromogenic Reagent Kit (Advanced Cell Diagnostics Inc.) was used according to the manufacturers’ protocol. A set of 14 pairs of PMCV probes ‘V‐piscine‐myocarditis‐ORF1’ (cat.no. 812021; Advanced Cell Diagnostics Inc.), targeting the PMCV capsid gene area at nt 1050–1757 bp on GenBank reference JQ728724.1 were designed by the manufacturer using custom software as described by (Wang et al., 2012). In addition, probes targeting salmon peptidylprolyl isomerase B (ppib, Cat. No. 494421) and bacterial dapB (dapB, Cat. No. 310043) were included during optimalization. The optimal concentration of PMCV probes was adjusted in a pilot study by testing a dilution series of the probe (1:1 to 1:4 by volume) in 1X RNAscope® Wash Buffer supplied with the kit, and the 1:2 dilution was selected. In short, deparaffination of non‐stained serial sections of selected samples prepared as described for histopathology was performed with xylene baths before several steps of rehydration in alcohol baths. Endogenous peroxidases in the rehydrated slides were blocked by hydrogen peroxidase treatment for 10 min, followed by 15‐min boiling of the sections in target retrieval buffer, before a 15‐min incubation at 40°C with a protease. The slides were then hybridized with the same amount of probe on each slide at 40°C for 2 h. After probe hybridization, the slides were incubated with signal amplifiers (AMP1 – AMP6) in sequences of duration times and at temperatures recommended by the manufacturer, including separate washing steps. Fast Red chromogen was used to visualize the hybridization signal, before counterstaining the slides using Mayer's haematoxylin (Chemi Teknik, Oslo, Norway), diluted in distilled water (1:1 by volume), and mounting with cover glasses and VectaMount (Vector Labs).

### 2.9 Graphics and statistical analysis

Statistical analysis and graph presentation were performed using GraphPad Prism 9.1.0 (GraphPad Software Inc.). Two‐way ANOVA followed by Tukey's multiple comparisons test was used to analyse statistically significant differences between real‐time PCR Ct values for each time point per tissue/blood/mucus sample and between sample types per time point. Due to the cut‐off value at Cq 40 for the real‐time PCR procedure, Cq 40 was used as the value for all negative samples and samples with Cq > 40 in the calculations and presentations. Correlation analyses were performed using Microsoft Excel.

## 3 RESULTS

The experimental CMS challenge of Atlantic salmon was set up to compare histopathological changes related to CMS with levels of PMCV‐specific RNA through PCR studies, in the three cardiac compartments atrium, ventricular spongiosum and ventricular compactum and also mid‐kidney and skin/muscle tissues, in the early‐phase post‐challenge. Parallel samplings of blood and mucus were also included for evaluation as non‐lethal samples and comparison against viral detection in the tissues. No mortalities, clinical signs of disease or observations of atypical signs of disease were observed during the experiment.

### 3.1 Autopsy

Average weight of the experimental fish was 48.1 g at mock vaccination (7 weeks days before the challenge), 94.1 g at 10 dpc and 129.1 g at 52 dpc, and they were in normal condition. Only a few fish had macroscopic findings of non‐relevant character to the results of the studies, with the exception of fish 8 at 52 dpc, which had an enlarged atrium.

### 3.2 Development of CMS shown by histopathology

To study the development of disease, six fish were subjected to histopathology studies at 0 dpc, and 15 fish at each of the other three samplings, with a few exceptions of tissues missing from the sample set (Table 1). HE‐stained tissue slides of heart (including all three compartments), mid‐kidney and skin/muscle were evaluated by light microscopy, and cardiac tissues were scored according to Table 2.

**FIGURE 1:**
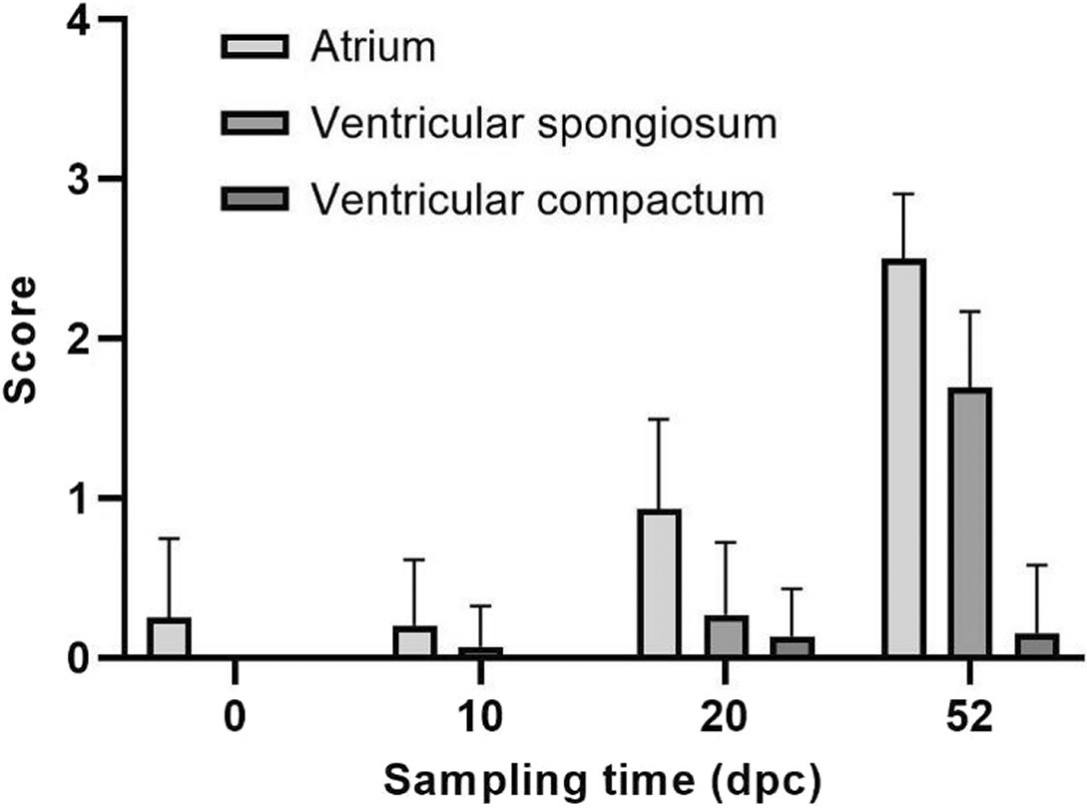
Histopathology scores of the three cardiac compartments atrium, ventricular spongiosum and ventricular compactum for all sampling time points, in accordance with Table 2. Average values plus standard deviation of the mean (SD) are shown (*n* = 5 at 0‐day post‐challenge (dpc), *n* = 15 at 10 and 20 dpc and *n* = 13 at 52 dpc)

**FIGURE 2:**
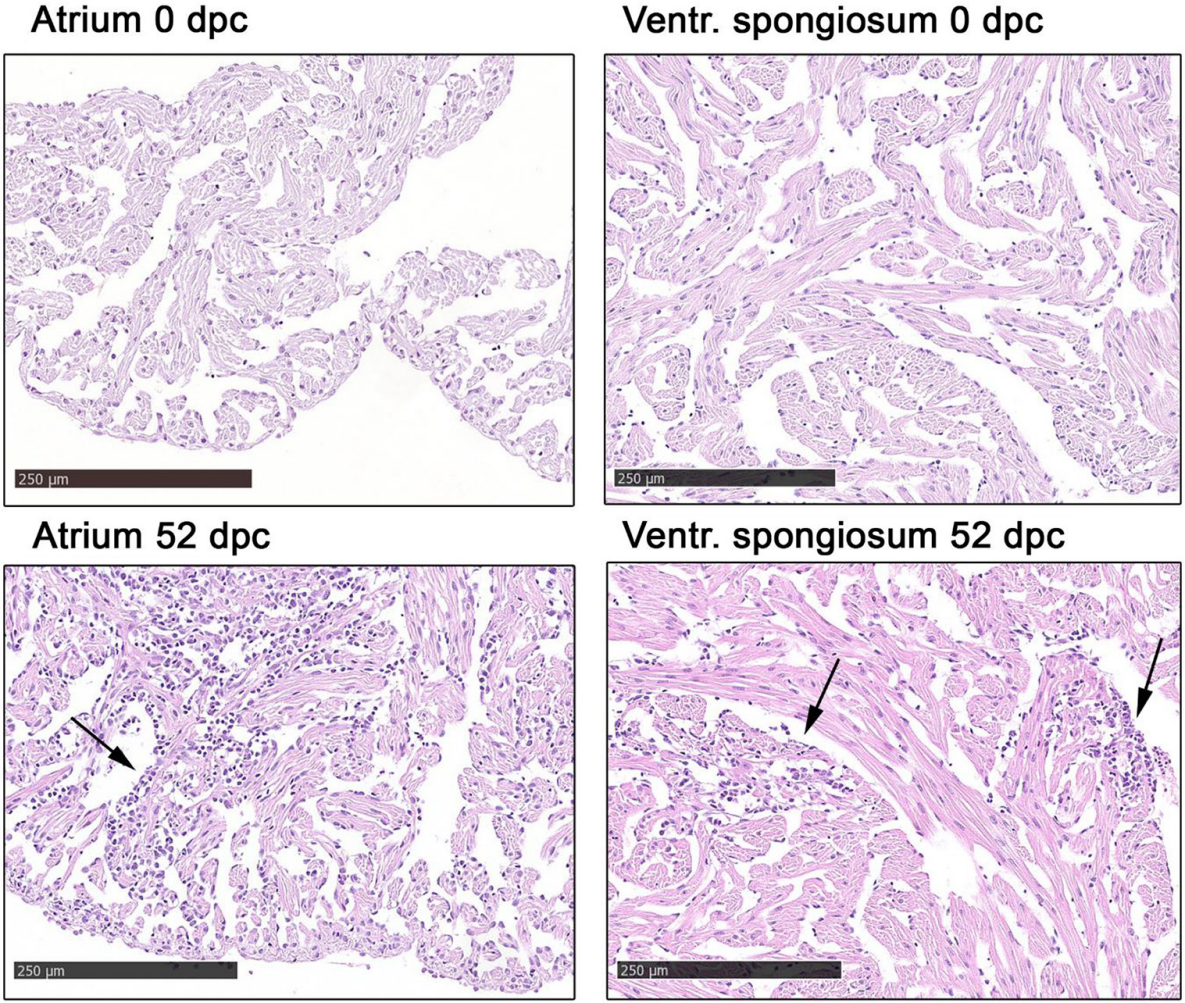
Normal atrium and ventricular spongiosum found at 0 dpc (exemplified by fish [F] 1 and 2, respectively), and typical CMS lesions (arrows) in atrium and ventricular spongiosum found at 52 dpc in F9 and 15, respectively. HE‐staining and standard light microscopy at 200x magnification (bar = 250 μm)

Before challenged (0 dpc), the majority of fish showed in general no signs of CMS‐related lesions (Figures 1 and 2), but a baseline finding of one or a few focal lesions of inflammatory cells in the epicardium was observed, and two fish had some lesions with very sparse inflammation of the atrium (Figure 1). All fish had moderate amounts of melanin and/or melanomacrophages in the interstitium of the kidney; most of them light eosinophilic foam‐like material in the urinary space of Bowman's capsule and eosinophilic material in lumen of a moderate number of renal tubuli. These were general findings in most fish at all sampling points, but a small increase in the amount of melanin and/or melanomacrophages in the kidneys were seen at the two last sampling points. In red skeletal muscle, about half the fish at the three first samplings presented with a sparse interfibrillar hypercellularity, which was observed in an increased number of fish at the last sampling.

At 10 dpc, the cardiac findings were comparable to the day of challenge, with exception of one individual with a sparse, focal lesion in the spongiosum of the ventricle. However, 10 days later, at 20 dpc, the number of individuals with sparse atrial lesions increased (Figure 1). The majority of these had a few focal lesions and an increased number of leukocytes (grade 1), but one individual had several distinct lesions also including a higher number of leucocytes (grade 2). A few fish also presented with a few focal lesions including increased number of leucocytes in the ventricular spongiosum. At this time point, sparse epicardial pathology was also apparent in the majority of fish, and five fish had several epicardial lesions, with a small to moderate increase in leukocyte number. Only one fish had sparse lesions of the compact ventricle at this point.

The most striking difference from the 20 dpc to the 52 dpc sampling was the increase in in number and severity of inflammatory lesions in atrial and ventricular spongiosum. Atrial changes particularly increased in severity, and ventricular lesions in number (Figure 2). All fish examined at this last sampling had several distinct or multifocal atrial lesions with a moderate to severely increased number of leukocytes. A majority of those with several distinct lesions had a tendency towards the more severe multifocal lesions (Figure 2). Furthermore, all fish had sparse or sparse to moderate lesions in the ventricular spongiosum. Epicardial changes were at the same, or slightly lower level, than at the 20 dpc sampling.Summarized, lesions of the atrium were recognized before lesions of the ventricular spongiosum. Indications of increasing inflammation in ventricular spongiosum were observed at 20 dpc, and this was more evident at 52 dpc. Epicardial lesions were mostly sparse, with a small increase from 10 to 20 dpc, continuing at the same low level at the last sampling. In general, very few and sparse pathological changes of the compact layer of the cardiac ventricle were observed: only a single fish at each of the two last samplings presented with few focal lesions and an increased number of leukocytes.

### 3.3 Presence of PMCV‐specific RNA in heart compartments, mid‐kidney and skin/muscle

**FIGURE 3:**
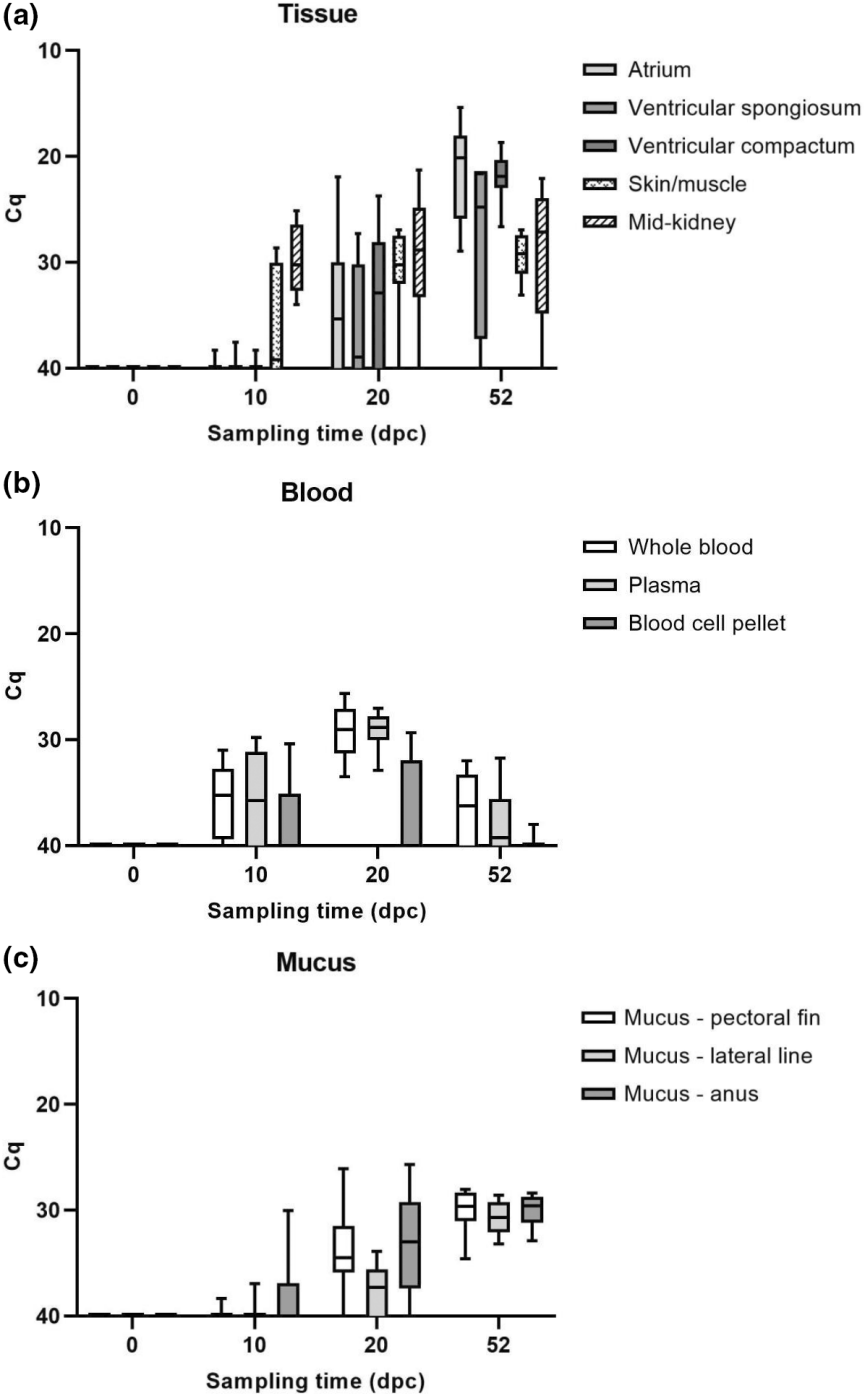
Levels of PMCV‐specific RNA given as Cq values, for all sampling time points, in days post‐challenge (dpc) in three heart compartments, mid‐kidney and skin/muscle and blood components and surface mucus. (a) Tissue samples of cardiac atrium, ventricular spongiosum, ventricular compactum, mid‐kidney and skin/muscle. (b) Parallel samples of blood: whole blood, plasma and blood cell pellet. (c) Mucus samples at three different locations of the surface of the fish: Pectoral fins, lateral line and anus. Results are presented as box plot with whiskers representing minimum and maximum values in the individual data set. *n* = 4 at 0 dpc and *n* = 15 at other time points, excluding ventricular spongiosum at 52 dpc where *n* = 4. Note the inverted numbering of the *Y*‐axis

The various tissues included for the detection of PMCV‐specific RNA revealed some variations in RNA levels in each individual over the course of early phases of infection (Figure 3a). At the earliest sampling after challenge (10 dpc), levels of PMCV RNA were highest in mid‐kidney (Figure 3a) and the RNA was detected with a 100% prevalence among the 15 individuals included. Viral RNA was also detected in skin/muscle tissue at a prevalence of 60%, although Cq values were generally high (spanning from 28.6 to 39.4). At this early time point, PMCV RNA was not detected in samples of any of the cardiac compartments, with the exception of a few samples with Cq value close to cut‐off (Figure 3a).

From 10 to 20 dpc, a significant increase in PMCV RNA load in the cardiac compartments atrium and ventricular compactum (*p* = .01–.05 and .001–.01, respectively) (Figure 3a) was seen, and a similar increase was also indicated for ventricular spongiosum. The resulting prevalence of detection is 60% of the individuals for each of all the three compartments separately. Still, detection of the virus RNA was not consistently distributed among compartments and individuals, and if any heart compartments are included per individual, the prevalence of detection is 80%. The levels of PMCV specific RNA in mid‐kidney samples were almost unchanged, and mid‐kidney was still the organ with most individuals with relatively high virus‐specific RNA levels (Cq around 30), but a wider inter‐individual variation in levels was seen, also including a few negative samples. Similar to the heart tissues, viral RNA detection also increased significantly in skin/muscle tissue (*p* = .01–.05) (Figure 3a) with resulting RNA levels higher, and with less inter‐individual variation, than each of the heart compartments and also higher prevalence for detection (86%). Both mid‐kidney and skin/muscle have significantly higher levels of viral RNA than ventricular spongiosum at this time point (*p* = .01–.05) (Figure 3a).

At the last sampling point (52 dpc), the distribution of highest PMCV RNA levels changed among the tissues. A significant increase (*p* < .0001) in the viral RNA levels was seen in atrium and ventricular compactum and probably also in ventricular spongiosum, but due to limited number of spongiosum samples (*n* = 4), statistical analyses for this cardiac compartment were not valid. At this point, all three heart compartments showed significantly higher PMCV RNA levels than mid‐kidney and skin/muscle (*p* < .0001 towards skin/muscle and *p* = .001–.01 towards mid‐kidney, statistics not valid for ventricular spongiosum due to limited number sampled at 52 dpc), while there was no significant change from 20 dpc in viral RNA levels neither in mid‐kidney nor skin/muscle samples.

There were no statistically significant differences between the Cq values obtained from the three heart compartments tested at each time point. Still, at individual levels there were variations, with correlation levels ranging from positive to non‐existent (Table 3), including strong correlation between atrium and spongiosum at 20 dpc. At 52 dpc, an almost perfect correlation is found (*r* = 0,98) between Cq values of spongiosum and compactum, but this may be related to the inclusion of only fish 1–4 as these are the only available from spongiosum at this time point.

**TABLE 3:** Correlation coefficients between Cq values of different cardiac compartments at the samplings 10, 20 and 52 dpc

Tissues	Correlation coefficient (*r*)
Atrium vs ventricular spongiosum 10 dpc	−0.02
Atrium vs ventricular spongiosum 20 dpc	0.78
Atrium vs ventricular spongiosum 52 dpc F1‐4	0.16
Atrium vs ventricular compactum 10 dpc	−0.07
Atrium vs ventricular compactum 20 dpc	0.61
Atrium vs ventricular compactum 52 dpc	0.42
Ventricular spongiosum vs compactum 10 dpc	−0.15
Ventricular spongiosum vs compactum 20 dpc	0.70
Ventricular spongiosum vs compactum 52 dpc F1‐4	0.98

An overview of all Cq data revealed that the highest individual sample levels of PMCV specific RNA in both mid‐kidney (Cq 21.3) and skin/muscle (Cq 27.0) were detected at 20 dpc, while all sampled cardiac compartments had highest individual RNA levels measured at 52 dpc (atrium Cq 15.4, compactum Cq 18.7 and spongiosum Cq 21.6, respectively). This is consistent with the overall trend observed, where the first significant increase in PMCV specific RNA levels appeared in the mid‐kidney, and, to some extent in the skin/muscle samples, at 10 dpc and then again at 20 dpc, before the cardiac compartments experienced a similar increase and reached the highest specific viral levels of the experiment at 52 dpc.

### 3.4 Presence of PMCV‐specific RNA in blood and mucus

Real‐time RT‐PCR detection of PMCV‐specific RNA was also used on samples of whole blood, plasma, blood cell pellet and mucus to evaluate these sample types as non‐lethal virus detection methods, and also, to study variation in viral presence in these samples over the time course of the initial phases of a PMCV infection.

#### 3.4.1 Blood

Heparinized whole blood and plasma samples had almost identical PMCV‐specific RNA levels at all three sampling points with in general low levels of PMCV RNA detected. Remarkably, from 10 to 20 dpc a significant increase in RNA levels was seen (*p* < .0001) and a 100% prevalence of detection was found, with very low inter‐individual variation for both sample types and relatively high RNA levels (Cq around or just below 30) compared with other samples at the same time point (Figure 3b). At 52 dpc, both whole blood and plasma PMCV‐specific RNA loads were significantly reduced (*p* < .0001), and the variation in viral RNA levels between individuals was increased, including a higher number of negative fish. The pelleted blood cells showed a similar, but statistically non‐significant trend, over time, with lower levels of PMCV RNA and a higher number of negative individual samples per sampling (Figure 3b).

#### 3.4.2 Mucus

At 10 dpc, the prevalence of individuals with at least one mucus sample with PMCV RNA detection was low (33.3%), and the resulting Cq levels were mainly close to cut‐off (Figure 3c). The levels increased significantly at 20 dpc (*p* < .0001, *p* = .001–.01 and *p* = .01–.05, respectively, for mucus of pectoral fin, lateral line and anus), where almost all fish had moderate‐to‐low amounts of PMCV‐specific RNA (Figure 3c), but with considerable inter‐individual variation for all samples, independent of sampling place. However, at the last sampling, all mucus samples were positive with a very low inter‐individual variation and general levels similar to the mid‐kidney samples at this time point (Figure 3c).

### 3.5 Immunohistochemistry – IHC

IHC was performed on 9 cardiac slides selected to represent all time points (Table 1). No specific staining was observed in any of the slides, except for the positive control, where a weak signal was seen in relation to a few atrial lesions, in the atrioventricular valves and in connective tissue at the interphase of ventricular compactum and ventricular spongiosum.

### 3.6 In situ hybridization

**FIGURE 4:**
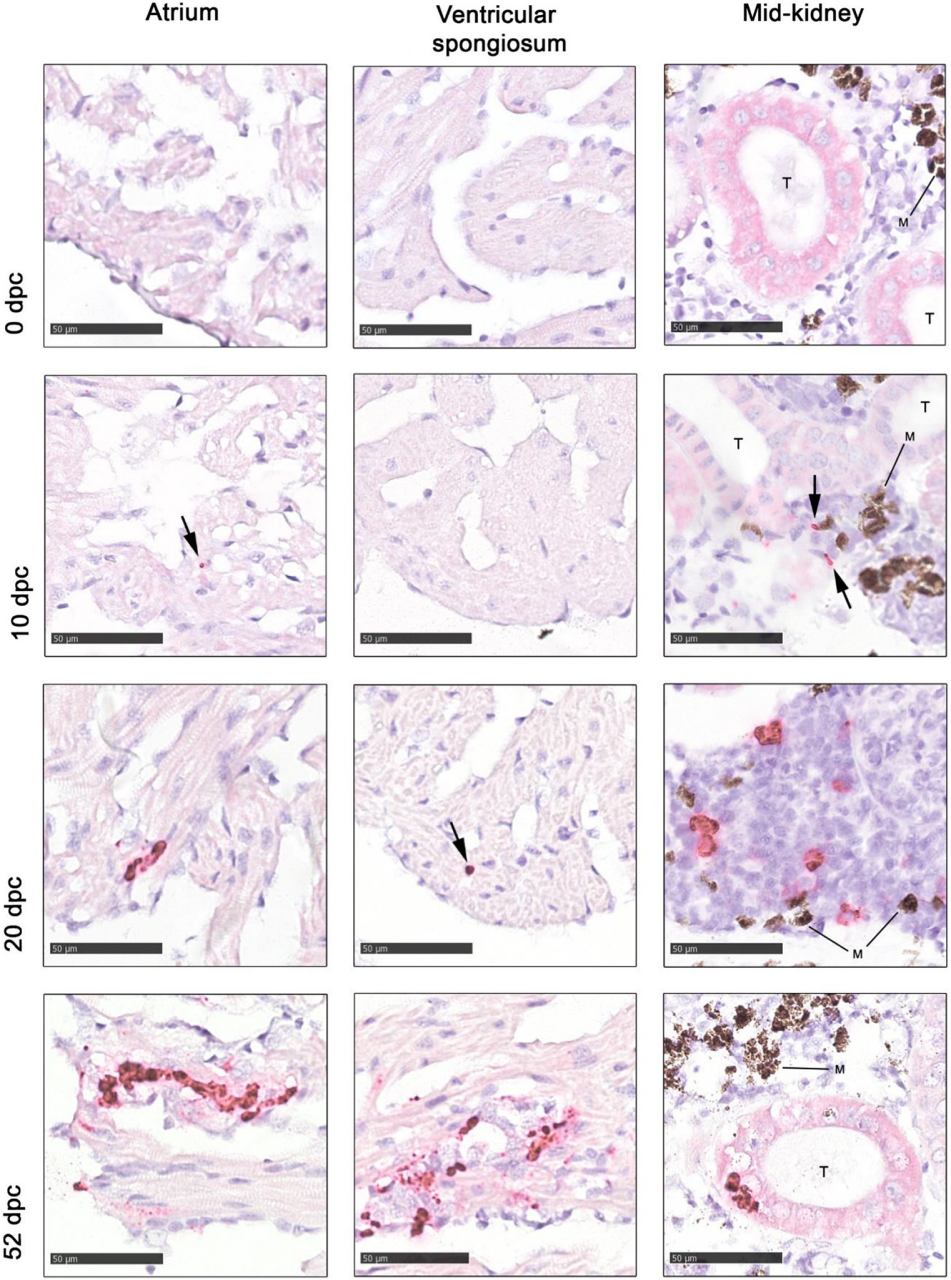
Examples from RNAscope in situ hybridization of cardiac atrium and ventricular spongiosum, represented by hearts of fish F1, F7, F2 and F1 at 0, 10, 20 and 52 dpc, respectively, and mid‐kidney represented by F3, F3, F2 and F2 at 0, 10, 20 and 52 dpc, respectively. Detection of PMCV‐specific RNA is visible as dark red staining (arrows in images of sparse staining). S = spongiosum, T = lumen of tubuli, M = melanin deposits and/or melanomacrophages in the interstitium of the kidney. All images 400× magnification, bar = 50 μm, standard light microscopy

An in situ hybridization procedure including a set of probes specific for PMCV ORF1 was established and optimized for evaluation as a diagnostic method, and to study the presence of PMCV specific RNA in relation to cardiac lesions and in mid‐kidney tissue over the time course of infection. Selected samples (Table 1) were subjected to the RNAscope in situ hybridization method and subsequently were examined by light microscopy for positive staining resulting from binding of the ORF1‐probes.Slides prepared from heart and mid‐kidney showed no positive staining for PMCV at 0 dpc (Figure 4). However, at 10 dpc, two very small foci of specific staining for PMCV were recognized in the atrium of one fish, in addition to very small, pinpoint staining of the nuclei of a few myocardial cells. Similarly, in the ventricle of the same fish, a few positive nuclear pinpoint signals were observed in apparently normal myocytes in one part of the compactum and the adjacent spongiosum, not related to inflammatory lesions. At the same time point, a few stained foci were observed in the interstitium of the mid‐kidney, probably cytoplasmatic, with indications of cytoplasmatic localization (Figure 4, mid‐kidney, 10 dpc).

**FIGURE 5:**
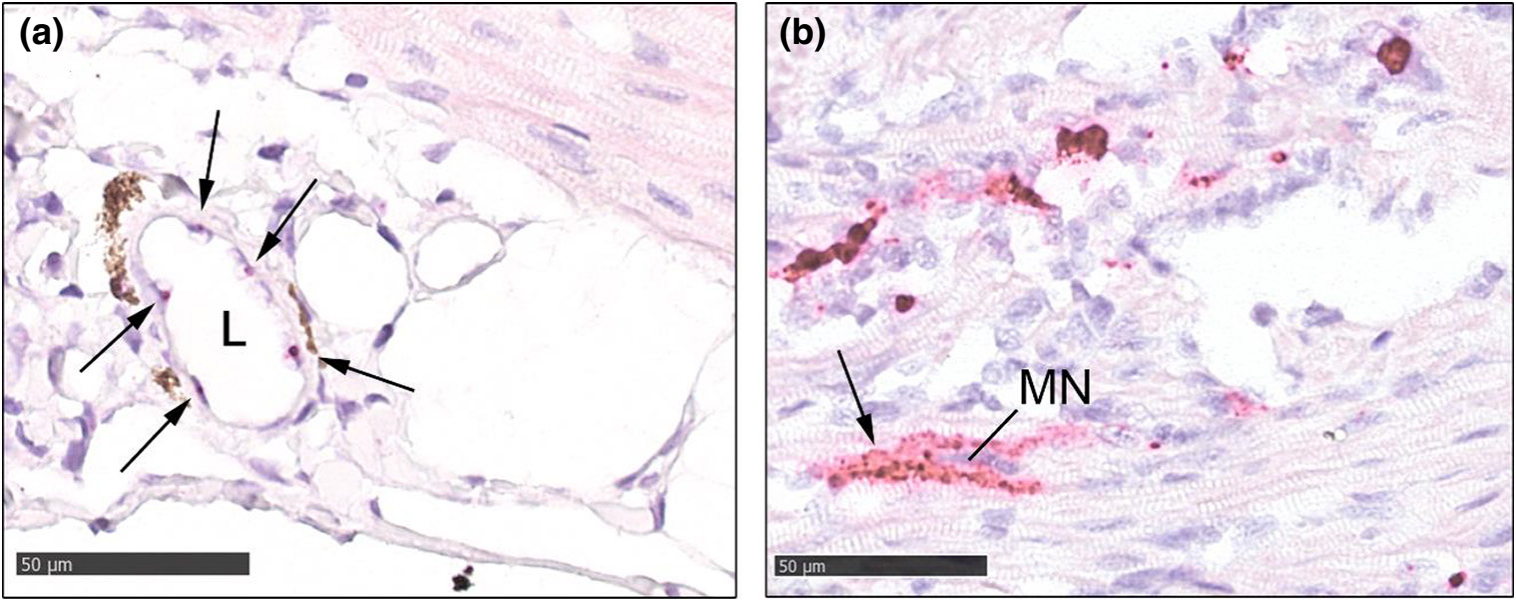
Details of RNAscope in situ hybridization results. Detection of PMCV‐specific RNA is visible as dark red staining. (a) Staining in endothelial nuclei in a coronary vessel (arrows). L = lumen of coronary vessel. (b) Staining (arrow) in atrial myocardial cells. Note the lack of staining in the myocardial nucleus (MN). Both images, 400 x magnification, bar = 50 μm, standard light microscopy

Ten days later (20 dpc), stronger positive signals were seen in the atrium of both examined fish (Figure 4), including three larger clusters of positive staining and one small cluster, respectively, with some relation to sparse inflammatory lesions. At this time point, pinpoint positive nuclear staining of various cells of both atrium and ventricle were also observed in endocardial, myocardial and endothelial cells (see Figure 5a). In the kidney, many distinct foci of positive staining were observed interstitially, mainly cytoplasmatic. In addition, some positive pinpoint nuclear staining of tubuli cells, as described for myocardial cells, was also observed, in contrast to almost no nuclear staining of interstitial cells at this time point (Figure 4).

Towards the end of the experimental period, at 52 dpc, there was abundant positive staining in, and related to, inflammatory foci of both atrium and ventricular spongiosum, with the most intense and widespread staining located in atriae, corresponding with the most severe cardiac lesions (Figures 1 and 4). The staining presented as clustered, strong signals in the middle of inflammatory lesions, more subtle and less intense positive staining of myocardium and surrounding tissue in areas of more sparse or moderate inflammation, in addition to moderate amounts of nuclear pinpoint staining in otherwise normal tissue of both compact and spongious layers of the heart. Severe cardiac lesions with strong specific PMCV RNA staining in cytoplasma showed no staining in myocardial nuclei (see Figure 5b). In this last sampling, most of the positive staining of mid‐kidney tissue were seen in tubuli cells, collecting tubuli and their nuclei, rather than in the interstitium as in the earlier samplings (Figure 4), as well as positive pinpoint nuclear staining of some glomeruli cells. However, a few positive signals could also be seen interstitially. A transition from strong cellular staining to more sparse nuclear pinpoint staining was observed as infection progressed.

## 4 DISCUSSION

Cardiomyopathy syndrome has since the first description in 1985 become a regular and relatively widespread disease in Atlantic salmon, in particular for the Norwegian, Scottish and Irish farming industry. In Norway, it has been ranked as the most important viral disease regarding both mortality and economic losses for the last years in Norway (Fritsvold & Jensen, 2020, 2021). Still, a decade after the viral agent was found (Haugland et al. (2011), Lovoll et al. (2010)), detailed knowledge on infection and pathogenesis, in particular in early phases, and a wide set of tools for diagnostic and research studies, are still lacking. Similar, there is also a need for a wider set of tools for diagnostic and research studies. Here, we have compared the detection of PMCV‐ and CMS‐related tissue lesions using previously used and well‐known diagnostic methods, that is, histopathology and real‐time RT‐PCR, to immunohistochemistry, an existing, but less used method, and the not previous published method RNAscope in situ hybridization. The methods were also used to study virus tropism by comparing PMCV‐specific RNA levels in a wider organ sample set, including non‐tissue sample types of various blood components and mucus.

Low or no mortality and almost complete absence of clinical signs typical for CMS is a common finding in CMS challenge trials of similar duration to this study (Haugland et al., 2011; Timmerhaus et al., 2011). Here, the only clinical sign observed was an enlarged atrium observed at autopsy 52 dpc. This was probably related to CMS, as histopathological cardiac lesions were sparse to moderate in all cardiac compartments, in combination with high levels of PMCV‐specific RNA load (Cq atrium 18.3). In general in this challenge, histopathological examinations confirmed CMS lesions characteristic for early‐phase infection, described as multiple, sparse foci of few inflammatory cells subendocardially in the atrium (Fritsvold et al., 2009; Haugland et al., 2011; Timmerhaus et al., 2011), and that these atrial lesions were recognized earlier than lesions in the spongiosum of the ventricle. Also, a typical development pattern of CMS lesions was seen over time in ventricular spongiosum, subsequent to the increased number and severity of atrial lesions. The compact layer had few and sparse lesions, in accordance with earlier descriptions (Bruno et al., 2013; Ferguson et al., 2006; Fritsvold et al., 2009; Haugland et al., 2011).Compared with PD and HSMI challenge trials (Christie et al., 2007; Kongtorp & Taksdal, 2009; Lund et al., 2016; Taksdal et al., 2015; Wessel et al., 2020), experimental CMS seems to have a slightly slower onset of infection (Fritsvold et al., 2009; Haugland et al., 2011; Timmerhaus et al., 2011), which probably also reflects the more protracted development seen in the field for CMS than in cases of, for example, PD (Graham et al., 2010; Kongtorp et al., 2004; McLoughlin & Graham, 2007; Taksdal et al., 2007). At the first sampling post‐challenge, 10 dpc, PMCV‐specific RNA was detected in highest concentrations in the mid‐kidney, while heart tissues, considered as the target organ of PMCV (Haugland et al., 2011; Timmerhaus et al., 2011), only showed detections close to cut‐off limit in a few samples. This might indicate that PMCV replicates and increases in concentration in kidney before it is transferred to the target organ, the heart. An early phase of viremia or primary viremia, where virus spreads from its initial infection site, is also supported by the increasing levels of virus RNA in blood samples, especially the ones including plasma.

The viremic phase seemed to continue towards the sampling ten days later, as the presence of viral RNA was increasing in all organs tested and in mucus and blood. The levels of PMCV RNA detected in plasma at this time point were remarkably high and consistent among the individuals, compared with levels and consistency among individuals in the other tissues tested, and give a strong support to an ongoing viremia at this stage of infection, although the exact time of the viremic peak cannot be decided as the sampling points included are too few. Cell damage is thought to trigger inflammation (Van Vleet & Ferrans, 1995), and increasing damage to infected cardiac cells was seen concurrently with increasing levels of PMCV RNA in the heart. These sparse early‐phase histopathological atrial lesions can be observed together with initial, sparse lesions in the spongy ventricle. The continuous high blood flow passing through the kidneys and the nature of basic renal functions, especially filtration, may explain the moderate‐to‐high levels of specific PMCV RNA in kidney samples during a viremia, as have been described previously in challenge trials (Timmerhaus et al., 2012).

At the last sampling point (52 dpc), the real‐time RT‐PCR results indicate that the viremia seen at 10 and 20 dpc was over. The relatively high and consistent levels of PMCV‐specific RNA in blood were reduced, including several samples where no virus RNA could be detected. At this time point, high levels of PMCV RNA in all cardiac tissues suggest that the virus probably has reached its target cells of the myocardium, where more efficient replication may take place, simultaneously inducing a phase of increasing inflammatory responses in the myocardium. Also interesting, concurrently with moderate virus RNA levels and high consistency among the individuals in skin/muscle at this time point, the mucus samples from both pectoral fin, lateral line and anus, all had their maximum levels of PMCV RNA in this sample set, also including very low inter‐individual variation. This could indicate that this stage of infection represents a phase where viral shedding from the fish through mucus is high. Shedding and horizontal transfer of the virus is supported by previous challenges including injection challenged fish transferring virus to cohabitants (Haugland et al., 2011). The time frame for such shedding to occur is not possible to define due to the limited sampling time points in this study and especially low frequency at this late time point. Similar studies on SAV have shown that the virus was present in mucus from 2 to 3 wpc and the presence continued for further 1 to 3 weeks, depending on virus subtype, and demonstrated that shedding and transmission of virus may occur through mucosal routes (Graham et al., 2011).

In addition to the expected strong positive staining shown in cardiac lesions at 52 dpc using the RNAscope in situ hybridization, a strong positive staining was detected in a few foci in the interstitium of mid‐kidney at 10 dpc, corresponding well with the increased levels of PMCV RNA shown in mid‐kidney at this early time point. The location of positive staining changed to kidney tubuli cells at 20 and 52 dpc, perhaps indicating a possible progression of the PMCV infection in the mid‐kidney: from initial infection of interstitial cells with haematopoietic function between sinusoids at the peak of the viremia, to cells of the excretory part of the kidney, most often in tubuli, but to some extent also in glomeruli, in the proposed post‐viremic phase. The RNAscope in situ hybridization was performed on 1 to 3 selected samples from each time point, representing the individuals with highest amounts of PMCV‐specific RNA in heart tissues, and does not necessarily represent the full sample set at each time point.

In some tissues and phases of the PMCV infection, very small, pinpoint positive staining was observed in the nuclei of apparently normal cells, in general not in relation to inflammatory or other pathological lesions. The pinpoint staining was found in nuclei of myocardial cells in cardiac spongiosum and compactum, in endothelium of coronary vessels, in mid‐kidney interstitial cells and tubuli cells. Replication of PMCV is described as cytoplasmatical (Haugland et al., 2011), and similar findings of strong specific PMCV RNA staining in cytoplasma and no staining in myocardial nuclei in relation to severe cardiac lesions found in our study support this. Also, the presence of a RNA virus, or viral genomic RNA fragments, in the nuclei of such cells is unexpected and should be investigated further to conclude the significance of the presence of PMCV RNA in the nuclei.

We have previously studied the detection of PMCV‐specific RNA in three heart compartments (atrium, ventricular spongiosum and ventricular compactum), in field samples from an ongoing CMS outbreak with a very high viral load. These results indicated that sampling from any of the three examined cardiac compartments would result in similar sensitivity of virus detection among the three heart compartments in similar late stages of CMS (Fritsvold et al., 2021). In this study, we included similar heart tissue compartment samples from individuals in early infection phases and with a lower viral load. Similar to the field study, there was no statistically significant difference between cardiac compartments over the samples set in PMCV RNA levels at any time points. Still, the results show that at individual level, the correlation of levels of PMCV RNA detected between the three included compartments was varying.

In this study, samples taken by non‐lethal methods were also included for comparison with a standard tissue sampling. Whole blood, plasma and pelleted blood cells from all individuals were tested for the presence of PMCV‐specific RNA. The results showed that moderate levels of PMCV RNA may be found in plasma, while the viral load in pelleted blood cells was low or non‐detectable at all time points. Also, similar levels were found in whole blood, but since the levels are comparable to plasma and pelleted cells almost negative, the presence found in whole blood is probably due to the plasma part and not the cells present. Mucus collected from pectoral fin, lateral line and anus reached similar detection levels as plasma samples, but in contrast, at late phase of infection.

For diagnostic purposes, heart has been the standard tissue for the detection of PMCV as virus‐specific RNA (Fritsvold & Jensen, 2021), and previous studies (Fritsvold et al., 2021; Timmerhaus et al., 2011) and present results confirm the heart as the organ of choice in active phases of PMCV infection with manifested cardiac inflammation and disease. Still, if the purpose is to screen for PMCV in early phases of an infection, the present results point towards a higher sensitivity of virus detection if kidney is chosen or included as sampled tissue. Also interesting, at 20 dpc in the present challenge, a comparable high sensitivity and low inter‐individual variation to the mid‐kidney were also found in plasma samples. However, compared with these results, mucus is probably not ideal for early‐phase screening purposes. Using non‐lethal blood sampling is beneficial to reduce loss for the farmer if screening is required for surveillance, and is also beneficial from an ethics perspective. More research is needed to confirm and optimize the high sensitivity using blood plasma samples to detect early PMCV presence in field cases.

As a consequence of an increasing number of field cases with double or even triple infections with PMCV, PRV‐1, ISAV or SAV, all causing heart pathology, assigning a diagnosis of CMS based on histopathology alone have become more challenging. As our results show, the real‐time RT‐PCR detects specific PMCV RNA from a very early infection stage, especially in kidney and plasma, and with high sensitivity throughout the infection. However, detection of PMCV specific RNA by this method may precede any specific CMS pathology and disease by weeks or even months (Svendsen et al., 2019; H. Sindre, pers.comm.). Although immunohistochemistry has been used to detect PMCV antigen in heart tissue with pathological changes (Fritsvold et al., 2021), our present results show that immunohistochemistry is dependent on a reliable primary antibody resulting in repeatable, sensitive and robust results to be suitable for diagnostic purposes.

In contrast, the RNAscope in situ hybridization method specifically detects PMCV‐specific RNA in connection with observed heart pathology, showing increased sensitivity and robustness compared with the immunohistochemistry. For implementation in routine diagnostics, the method should be further validated with focus on specificity, including testing of fish with other relevant cardiac diseases and mixed infections. Fully validated, the RNAscope ISH method may be a valuable supplement to histopathology to provide reliable CMS diagnoses in situations where the pathological changes are uncharacteristic or mixed.

In conclusion, this study adds knowledge on how PMCV infection progress in individual fish in the early phases of CMS. Our results indicate a viremic phase of PMCV at 10 to 20 dpc, preceding the cardiac lesions. In this early phase, virus could also be detected in relatively high amounts in mid‐kidney, first in interstitial cells, then later also in tubuli cells and cells of collecting tubuli. Plasma and/or mid‐kidney samples may, therefore, be candidates to screen for early‐phase PMCV infection. Secondly, our results support the heart as the organ of choice for both viral detection and histopathological diagnosis when cardiac lesions have emerged. In addition, the RNA scope in situ method has the potential to become a very useful diagnostic tool, both in pathogenesis studies and for routine diagnostics.

## CONFLICTS OF INTEREST

There are no conflicts of interest declared by the authors.

## Data Availability

The data that support the findings of this study are available from the corresponding author upon reasonable request.
